# Akrinor^TM^, a Cafedrine/ Theodrenaline Mixture (20:1), Increases Force of Contraction of Human Atrial Myocardium But Does Not Constrict Internal Mammary Artery *In Vitro*

**DOI:** 10.3389/fphar.2017.00272

**Published:** 2017-05-23

**Authors:** Benjamin Kloth, Simon Pecha, Eileen Moritz, Yvonne Schneeberger, Klaus-Dieter Söhren, Edzard Schwedhelm, Hermann Reichenspurner, Thomas Eschenhagen, Rainer H. Böger, Torsten Christ, Sebastian N. Stehr

**Affiliations:** ^1^Department of Experimental Pharmacology and Toxicology, University Medical Center Hamburg-EppendorfHamburg, Germany; ^2^Department of Cardiovascular Surgery, University Medical Center Hamburg-EppendorfHamburg, Germany; ^3^Department of Clinical Pharmacology and Toxicology, University Medical Center Hamburg-EppendorfHamburg, Germany; ^4^Department of Anesthesia and Critical Care Medicine, Leipzig UniversityLeipzig, Germany

**Keywords:** hypotension, intraoperative, catecholamines, ephedrine, indirect sympathomimetics, phosphodiesterase-inhibitor, α-adrenoceptors, β-adrenoceptors

## Abstract

**Background:** Intraoperative hypotension is a common problem and direct or indirect sympathomimetic drugs are frequently needed to stabilize blood pressure. Akrinor^TM^ consists of the direct and the indirect sympathomimetic noradrenaline and norephedrine. Both substances are covalently bound to the phosphodiesterase (PDE) inhibitor theophylline, yielding theodrenaline and cafedrine, respectively. We investigated pharmacodynamic effects of Akrinor^TM^ and its constituents on contractile force and tension in human atrial trabeculae and internal A. mammaria rings.

**Methods:** Isometric contractions were measured in human atrial trabeculae at 1 Hz and 37°C. CGP 20712A and ICI 118,551 were used to elaborate β_1_- and β_2_-adrenoceptor (AR) subtypes involved and phenoxybenzamine to estimate indirect sympathomimetic action. PDE-inhibition was measured as a potentiation of force increase upon direct activation of adenylyl cyclase by forskolin. Human A. mammaria preparations were used to estimate intrinsic vasoconstriction and impact on the noradrenaline-induced vasoconstriction.

**Results:** Clinically relevant concentrations of Akrinor^TM^ (4.2–420 mg/l) robustly increased force in human atrial trabeculae (EC_50_ 41 ± 3 mg/l). This direct sympathomimetic action was mediated via β_1_-AR and the effect size was as large as with high concentrations of calcium. Only the highest and clinically irrelevant concentration of Akrinor^TM^ increased the potency of forskolin to a minor extent. Norephedrine has lost its indirect sympathomimetic effect when bound to theophylline. Increasing concentrations of Akrinor^TM^ (4.2–168 mg/l) alone did not affect the tension of human A. mammaria interna rings, but shifted the noradrenaline curve rightward from -logEC_50_ 6.18 ± 0.08 to 5.23 ± 0.05 M.

**Conclusion:** Akrinor^TM^ increased cardiac contractile force by direct sympathomimetic actions and PDE inhibition, did not constrict A. mammaria preparations, but shifted the concentration-response curve to the right, compatible with an α-AR antagonistic effect or PDE inhibition. The pharmacodynamic profile and potency of Akrinor^TM^ differs from noradrenaline and norephedrine *in vitro*. We anticipate metabolism of theodrenaline and cafedrine resulting in a different pharmacodynamic profile of Akrinor^TM^ *in vivo*.

## Introduction

Intraoperative hypotension is a common event that has an important negative influence on hospital stay and mortality in patients undergoing surgery (Sessler et al., 2012). Multiple pharmacological agents are available to treat intraoperative hypotension that target the sympathetic system (Gamper et al., 2016).

One of the agents most commonly used in Germany is Akrinor^TM^, a mixture of sympathomimetic agents, i.e., noradrenaline and norephedrine, both covalently bound to theophylline, yielding theodrenaline and cafedrine, respectively. Akrinor^TM^ is used for decades in clinical practice in Germany (Heller et al., 2015). It is recommended as an anti-hypotensive agent by the German Society of Anesthesiology and Intensive Care Medicine guidelines for anesthesia-related hypotension in parturients (Marcus et al., 2011; Heesen and Veeser, 2012). Studies focusing on pharmacodynamics of Akrinor^TM^ are sparse. Most of them were done in *in vivo* animals or human experiments. In regard to its constituents one would expect mixed direct/indirect sympathomimetic effects from Akrinor^TM^, reinforced by PDE-inhibition. Cardiovascular effects of Akrinor^TM^ were reviewed recently in this journal (Bein et al., 2017). In summary, Akrinor^TM^ has a positive inotropic and chronotropic effect in humans (Sternitzke et al., 1984; Heller et al., 2015). In contrast to other vasopressor agents, systemic vascular resistance remains almost unchanged (Sternitzke et al., 1984). As a result, MAP increases by about 10 mmHg within 5 min after *i.v.* injection. Maximum increase in MAP was reached 17 min after the intravenous application. In patients undergoing general anesthesia the ED_50_ to achieve a 10% increase in MAP within 5 min was 1.5 ± 0.08 mg/kg body weight (Heller et al., 2015). Maximum recommended single bolus dose for an intravenous injection is one ampoule of Akrinor^TM^, consisting of 200 mg cafedrine hydrochloride and 10 mg theodrenaline hydrochloride (Bein et al., 2017). We therefore transferred a typical clinical scenario, e.g., *i.v.* injection of one ampoule of Akrinor^TM^ to an experimental setting. Yet, it remains unclear to what extent every single pharmacodynamic mechanism contributes to the overall effect of Akrinor^TM^. Therefore, we aimed to investigate how conjugation of theophylline to noradrenaline and norephedrine alters pharmacodynamics.

Here, we investigated the effects of Akrinor^TM^ pharmacodynamics in comparison to the effects of noradrenaline, norephedrine, and theophylline on force generation in isolated human atrial trabeculae and on tension in human IMAs.

## Materials and Methods

Right atrial appendages and segments of left and right IMA were obtained during open-heart surgery at the University Heart Centre Hamburg. The study followed the declaration of Helsinki. All patients gave written informed consent. According to the guidelines of the ethical review committee Hamburg, Germany, there is no need for an approval in this case. Patient data were used anonymized. Exclusion criteria were intermittent or chronic atrial fibrillation, inability of patients to give informed consent or treatment with Akrinor^TM^ before the operation. After excision, right atrial appendages were immediately placed at room temperature into a non-oxygenated cardioplegic solution [in mM: NaCl 100, taurine 50, glucose 20, KCl 10, MgS0_4_ 5, MOPS (3-(*N*-morpholino)propanesulfonic acid) 5, KH_2_PO_4_ 1.2] containing 30 mM of the myosin ATPase inhibitor BDM (2,3-butanedione monoxime) and transferred to the laboratory in less than 10 min. IMA preparations were transported in Tyrode’s solution (see below).

### Force Measurements in Right Atrial Appendages

Up to eight trabeculae were dissected from one appendage. Experiments were performed in modified Tyrode’s solution containing (mM): NaCl 126.7, KCl 5.4, CaCl_2_ 1.8, MgCl_2_ 1.05, NaH_2_PO_4_ 0.42, NaHCO_3_ 22, EDTA 0.04, ascorbic acid 0.2 and glucose 5.0. The solution was maintained at pH 7.4 by bubbling with a mixture of 5% CO_2_ and 95% O_2_. Atrial trabeculae were mounted in pairs, attached to SWEMA 4–45 strain gauge transducers in an apparatus containing above solution at 37°C and paced at 1 Hz. Trabeculae were pre-stretched to 50% of the length associated with maximum developed force. Sample sizes were chosen based on previous experience with experiments with positive inotropic substances and concomitant availability of myocardial and vascular preparations. The trabeculae were distributed randomly in four organ baths, usually two per bath. The assignment of experimental groups to the baths was randomized by drawing lots. In all experiments, unless otherwise indicated, we followed a protocol aimed to minimize effects of endogenous catecholamines. To this end, tissues were incubated with 6 μM phenoxybenzamine for 90 min. Phenoxybenzamine is an unselective α-adrenoceptor antagonist and increases release of noradrenaline (Enero et al., 1972). Trabeculae were washed from released catecholamines and let stabilized over additional 30 min. Force was recorded using Chart Pro for Windows version 5.51 analysis program (ADI Instruments, Castle Hill, NSW, Australia).

### Tension Measurements in Rings Prepared from Internal Mammaria Arteries

The adherent connective tissue was carefully dissected, and the artery was cut in up to eight rings of 3 mm width. The IMA segments were suspended on wire hooks in the organ bath described above (same Tyrode’s solution). Resting tension was increased stepwise from 2 up to 20 mN (four steps, every step lasts 7 min). KCl (100 mM) was applied and washed out six times to confirm proper function of the vessel rings. All experiments with IMA segments were performed in the absence of phenoxybenzamine.

### Drugs and Chemicals

Akrinor^TM^ is a mixture of cafedrine hydrochloride (200 mg) and theodrenaline hydrochloride (10 mg) in a 2 ml solution. Pharmacologically relevant concentrations were estimated at 42 mg/l (based on an injection of a single ampoule Akrinor^TM^ assuming 5 l blood volume). 42 mg/l Akrinor^TM^ contain 5.7 μM noradrenaline conjugated to theophylline, 101 μM norephedrine conjugated to theophylline, and 106.7 μM conjugated theophylline. Therefore, we performed experiments with theophylline at concentrations of 10, 100, and 1000 μM to compare effects of 4.2, 42, and 420 mg/l Akrinor^TM^. Akrinor^TM^, theophylline, norephedrine, and cafedrine were provided by TEVA ratiopharm (Ulm, Germany). Noradrenaline, phenoxybenzamine, forskolin, CGP 20712A (2-hydroxy-5-[2- [[2-hydroxy-3-[4-[1-methyl-4-(trifluorometyl)-1H-imidazol-2- yl]phenoxy]propyl]amino]ethoxy]-benzamide), ICI 118,551 (1-[2,3-dihydro-7-methyl-1H-inden-4-yl]oxy-3-[(1-methylethyl) amino]-2-butanol) and all other chemicals were obtained from Sigma-Aldrich (Darmstadt, Germany).

### Statistics

Data are expressed as mean ± SEM. When more than one tissue from a patient was available for one experimental group, mean values were calculated for individual patients. LogEC_50_ were obtained by fitting sigmoidal concentration-response curve to data points from individual experiments. Paired *t*-test was used to compare logEC_50_ values under PDE-inhibition to the respective controls obtained from the same patients. If more than two experimental groups were present, we compared maximum effects and logEC_50_ values by one-way ANOVA followed by Bonferroni *post hoc* test. Curve fitting and all statistics were done by Prism GraphPad 5.0 (La Jolla, CA, United States).

## Results

### Effects of Akrinor^TM^ on Force in Human Atrial Trabeculae are Mediated via β_1_-AR

**FIGURE 1:**
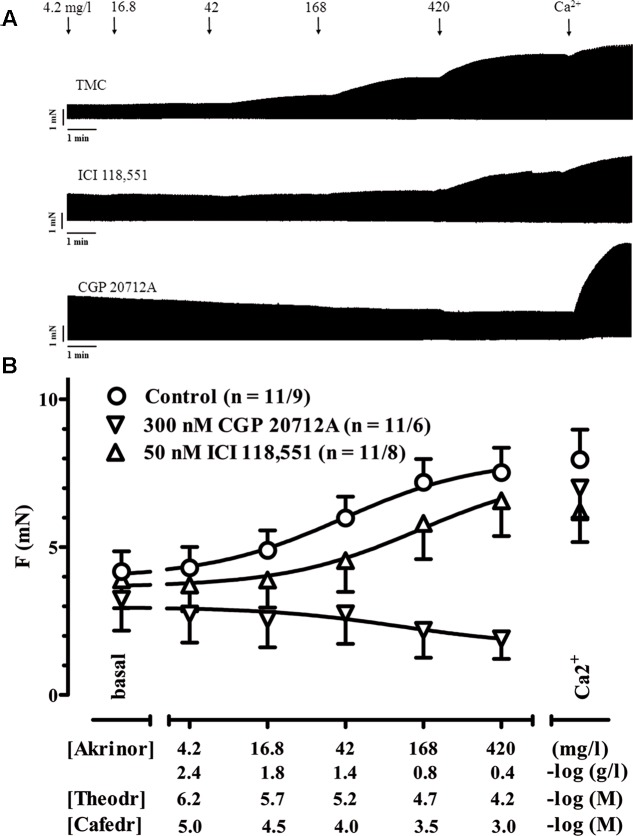
**Positive inotropy by Akrinor^TM^: involvement of β-AR subtypes. (A)** Original registrations of force in human atrial trabeculae exposed to increasing concentrations of Akrinor^TM^ under control condition and in the presence of 300 nM CGP 20712A to block β_1_-AR or 50 nM ICI 118,551 to block β_2_-AR. **(B)** Mean values ± SEM of force under basal condition (Ba, in the presence of the respective β adrenoceptor antagonists or controls) and after exposure to increasing concentrations of Akrinor^TM^ expressed in mg/l and in –log (g/l). Concentrations of the two constituents of AkrinorTM, theodrenaline (Theodr) and cafedrine (Cafedr) are given in –log (M). Maximum inotropic response was assessed by the addition of 8 mM Ca^2+^ at the end of the experiments. Experiments were performed in the presence of 6 μM phenoxybenzamine. n/n, indicates number of trabeculae/number of patients. Decrease in force for Akrinor^TM^ in the presence 300 nM CGP 20712A is not different from TMCs (data not shown).

In a first set of experiments, we investigated if Akrinor^TM^ evokes a positive inotropic effect in human atrial trabeculae via stimulation of β-adrenoceptors (AR). Experimental concentrations were varied from 4.2 up to 420 mg/l in a cumulative manner in order to construct concentration-response curves. We measured Akrinor^TM^ effects in the presence of β-AR subtype selective antagonists to elucidate the involvement of β-AR subtypes. CGP 201712A (300 nM) was used to block β_1_-AR and ICI 118,551 (50 nM) to block β_2_-AR. Proper inotropic reaction of the muscles was confirmed at the end of each experiment by increasing Ca^2+^ concentration from 1.8 to 8 mM to provoke maximal inotropic responses (**Figures 1A,B**).

Akrinor^TM^ already increased force of contraction at concentrations 10-fold lower than expected from intravenous injection of a single ampoule Akrinor^TM^ (4.2 mg/l). Maximum responses were reached at concentrations of 420 mg/l and were not smaller than effects of high calcium concentration indicating full agonist activity of Akrinor^TM^. The presence of the β_1_-AR antagonist CGP 201712A (300 nM) completely blunted Akrinor^TM^ inotropic effects, while responses to Ca^2+^ were preserved. The lack of any CGP 20712A-resistent positive inotropy indicates exclusive mediation of Akrinor^TM^ effects via β_1_-AR. ICI 118,551 shifted the concentration-response curve from an EC_50_ value of 40.8 ± 3.1 mg/l to 142 ± 4.4 mg/l (*p* < 0.01; *n* = 11/9 vs. 11/8, unpaired *t*-test). Effects of ICI118,551 further substantiate the latter interpretation. The small shift cannot be interpreted as a β_2_-AR-contribution to Akrinor^TM^ positive inotropic effect. ICI 118,551 preferentially binds to β_2_-AR and 50 nM ICI 118,551 should shift the curve for β_2_-AR-mediated effects more than two log units. However, there is also some affinity of ICI 118,551 to β_1_-AR. This small shift of the Akrinor^TM^ concentration-response curve by ICI 118,551 fits nicely to the known affinity data of ICI 118,551 to β_1_-AR and is in line with recently measured dose shift in β_2_-AR-KO mice (Pecha et al., 2015).

### Only Clinically Irrelevant High Concentrations of Akrinor^TM^ Potentiate Forskolin Effects on Force

**FIGURE 2:**
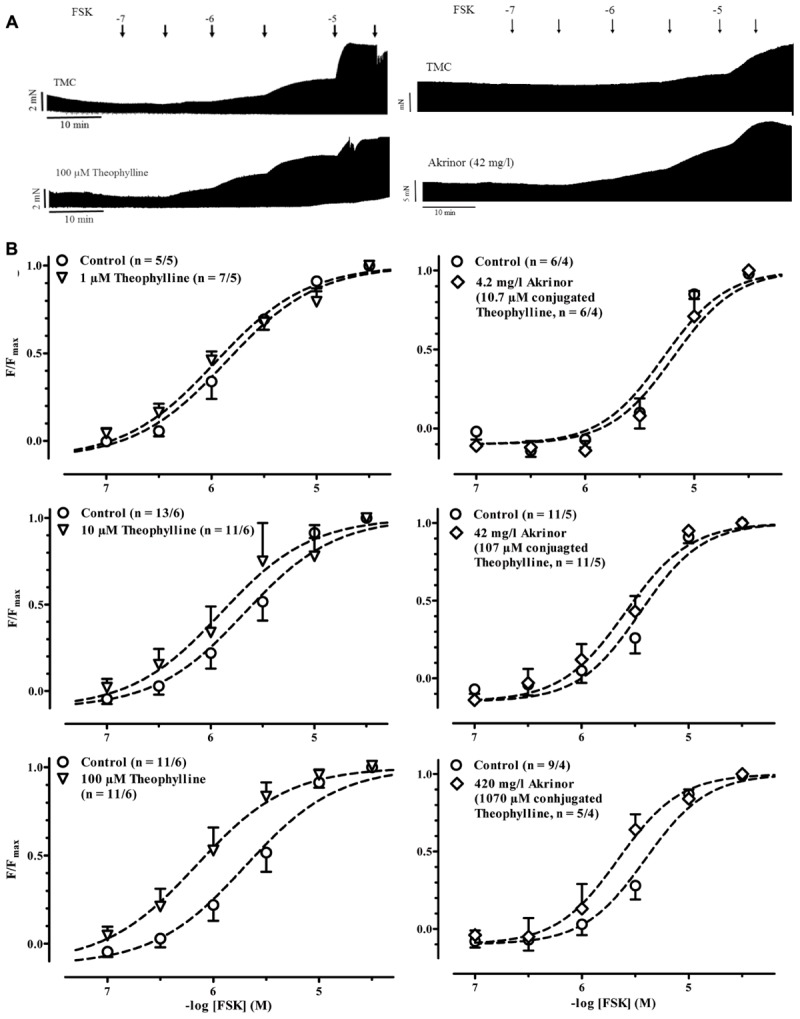
**Potentiation of forskolin inotropy by theophylline and Akrinor^TM^. (A)** Original registrations of force in human atrial trabeculae exposed to increasing concentrations of forskolin (FSK) under control condition (CGP 20712A present) and in the presence of 100 μM theophylline (left) and 42 mg/l Akrinor^TM^ (right). Respective TMC exposed to FSK only are given on top. **(B)** Normalized force responses to FSK in the presence of different concentration of theophylline (left) and Akrinor^TM^ (right). For absolute force data see **Supplementary Figure S1**. Because at low concentrations notorious decline in force dominates, negative values result for low (ineffective) concentrations of FSK. Experiments were performed in the presence of 6 μM phenoxybenzamine.

In a next set of experiments, we examined if Akrinor^TM^ exhibits PDE-inhibition in human atrial trabeculae. The classical approach to measure PDE-inhibition is to determine the leftward shift of the concentration-response curve for the inotropic effects of catecholamines (Rall and West, 1963). Since Akrinor^TM^ directly activates β_1_-AR, we needed to modify the protocol and performed all the following experiments in the presence of the β_1_-AR antagonist CGP 20712A (300 nM). To activate cAMP production we used the direct adenylyl cyclase activator FSK. FSK produced the expected positive inotropic effect in control experiments and in the presence of different concentrations of theophylline and Akrinor^TM^ (**Figure 2A**). To make changes in sensitivity more clear we normalized force responses to its individual maximum (**Figure 2B**). In order to minimize data scattering based on variability between patients we compared data obtained with theophylline or Akrinor^TM^ to untreated trabeculae from the same patient as controls. Therefore, a paired *t*-test was used to compare the effect of the intervention. First, we checked for a leftward shift of the concentration-response curve for the positive inotropic effect of forskolin by the prototypical PDE-inhibitor theophylline. 1 μM theophylline did not shift the concentration-response curve for subsequent exposure to forskolin. There was a trend with 10 μM theophylline. However, 100 μM theophylline were necessary to shift the FSK concentration-response curve by half a log unit from -logEC_50_ of 5.52 ± 0.14 to 6.01 ± 0.2 M (*p* < 0.001, *n* = 11/6 each group, paired *t*-test). Next, we repeated experiments with Akrinor^TM^. The sample size for experiments with AkrinorTM was adapted to experiments necessary to confirm theophylline-induced leftward-shift of concentration-response curve for forskolin. Only very high, clinically irrelevant concentrations of Akrinor^TM^ (420 mg/l) produced significant potentiation of FSK effects, from -logEC_50_ value of 5.36 ± 0.31 to 5.7 ± 0.38 M (*p* < 0.05, *n* = 9/4 vs. 4/4, paired *t*-test), conceivable by PDE-inhibition. On the other hand, a matching concentration of theophylline (1 mM) alone generated a sustained increase in force, but FSK effects were blunted (**Supplementary Figure S1** and Table S1). We would interpret this finding as an indication of theophylline toxicity.

### Blunted Indirect Sympathomimetic Effects by Cafedrine on Heart Muscle

**FIGURE 3:**
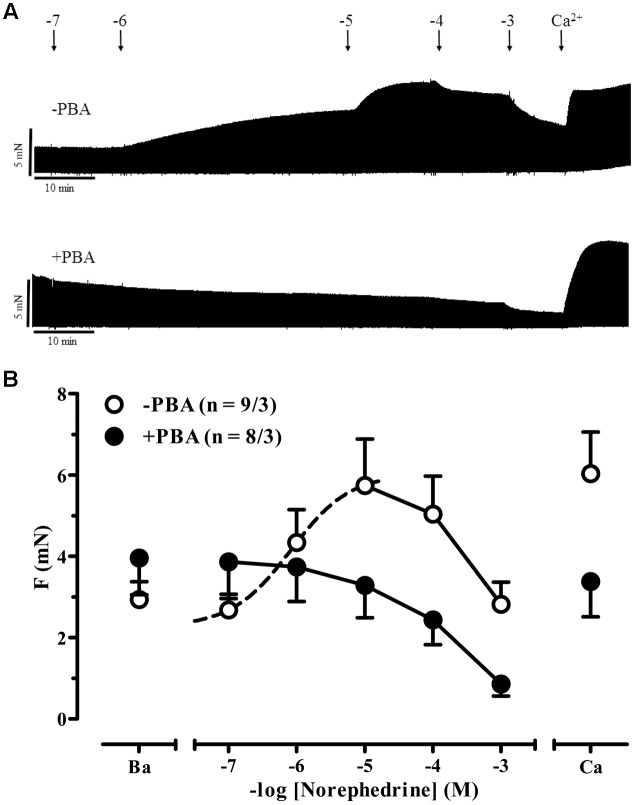
**Indirect sympathomimetic effects of norephedrine. (A)** Original registrations of force in human atrial trabeculae exposed to increasing concentrations of norephedrine under control condition (no phenoxybenzamine, -PBA) and in the presence of 6 μM phenoxybenzamine (+PBA). **(B)** Mean values ± SEM of force under basal condition **(B)** and after exposure to increasing concentrations of norephedrine under control conditions (Control) and in the presence of 6 μM phenoxybenzamine (PBA). Maximum inotropic response was assessed by the addition of 8 mM Ca^2+^ at the end of the experiments. n/n indicates number of trabeculae/number of patients. Dotted line indicates sigmoidal curve fit. Solid lines indicate negative inotropy by very high concentrations of norephedrine.

**FIGURE 4:**
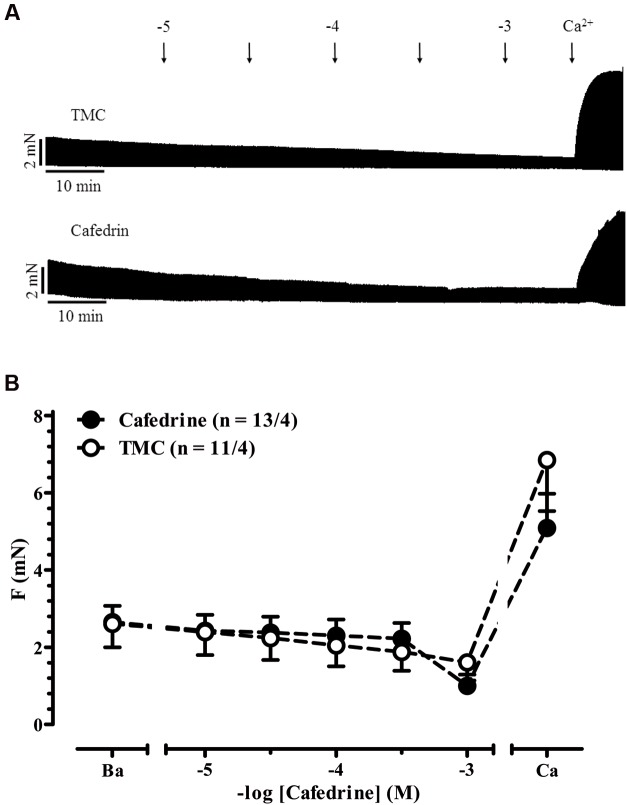
**Indirect sympathomimetic effects of cafedrine. (A)** Original registrations of force in human atrial trabeculae exposed to increasing concentrations of cafedrine and in a TMC. **(B)** Mean values ± SEM of force under basal condition **(B)** and after exposure to increasing concentrations of cafedrine. Maximum inotropic response was assessed by the addition of 8 mM Ca^2+^ at the end of the experiments. n/n, indicates number of trabeculae/number of patients. Experiments were performed in the absence of phenoxybenzamine.

Akrinor^TM^ contains theodrenaline and cafedrine. The latter drug is a conjugate of theophylline and norephedrine. Norephedrine is known to induce indirect sympathomimetic effects releasing noradrenaline from nerve endings (Liebmann, 1961; Trendelenburg et al., 1962). Therefore, we examined if an indirect sympathomimetic effect of cafedrine may contribute to the overall effects of Akrinor^TM^. Classification of sympathomimetic effects as “indirect” critically depends on demonstration that catecholamine stores involved (Trendelenburg, 1969). For this purpose, we used phenoxybenzamine, a drug that non-selectively blocks α-AR (Langer, 1977). Micromolar concentrations of phenoxybenzamine inhibit neuronal uptake of noradrenaline by more than 50% (Enero et al., 1972). Therefore, we compared norephedrine effects in the absence and presence of phenoxybenzamine (**Figures 3A,B**). In the absence of phenoxybenzamine norephedrine increased force significantly from 2.9 ± 0.4 to 5.7 ± 1.1 mN (*p* < 0.05, *n* = 9/3, paired *t*-tests) with a calculated -logEC_50_ of 6.1 M. This effect was completely abolished by phenoxybenzamine. It should be noted that under both conditions a clear negative inotropic effect occurred at concentrations >10 μM, indicating some toxic effects of norephedrine not related to its indirect sympathomimetic effects. In contrast to norephedrine, cafedrine did not show any positive effect (**Figures 4A,B**). Decline in force in cafedrine-treated trabeculae was not significantly different from TMC. Taken together our data suggest that the (indirect) sympathomimetic effect of norephedrine is lost when conjugated to theophylline.

### Effects of Akrinor^TM^ and Its Constituents on Basal and Noradrenaline Increased Tone in Human Arteries

Blood pressure is the product of cardiac output and vascular resistance. Any increase in blood pressure can result from one or more elements of positive inotropy, positive chronotropy, or vasoconstriction. Here, we found three pharmacodynamic pathways that could explain *in vivo* effects of constituents of Akrinor^TM^ and norephedrine, eventually metabolized *in vivo*: direct and indirect sympathomimetic actions and potentiation of cAMP-evoked inotropy by PDE-inhibition. All three mechanisms involve β-AR in the myocardium. In contrast, α-AR mediate vasoconstriction in response to sympathetic nervous stimulation and therefore vascular resistance, while β_2_-AR-stimulation has opposing effects. In order to estimate whether arterial vasoconstriction contributes to Akrinor^TM^’s blood pressure rising effects we measured the effects of Akrinor^TM^ and some of its constituents on tension of human arterial rings. In addition, we estimated the impact on vasoconstriction evoked by the natural agonist on α-AR, noradrenaline.

**FIGURE 5:**
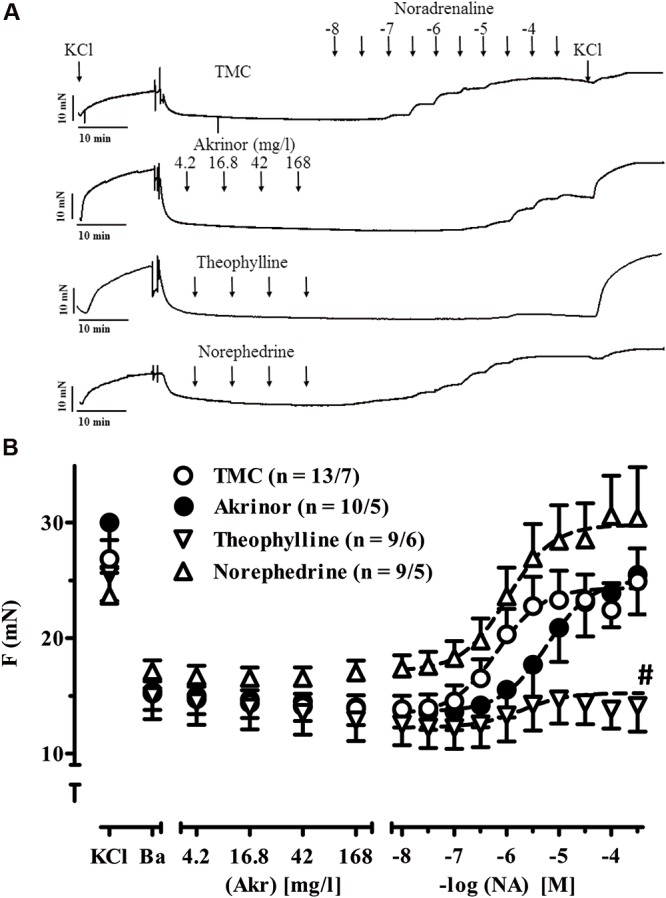
**Intrinsic effects of Akrinor^**TM**^ and its constituents on arterial tension and noradrenaline-induced vasoconstriction. (A)** Original registrations of tension in human IMA. Maximum tension was estimated by exposure to 100 mM KCl (KCl) first. After wash-out of the rings basal tension was assessed (Ba) and rings were exposed to increasing concentrations of Akrinor^TM^, theophylline, norephedrine or served as TMC. All preparations were subsequently exposed to noradrenaline in the continuous presence of the highest concentration of Akrinor^TM^, theophylline and norephedrine, respectively. **(B)** Mean values ± SEM of tension under basal condition **(B)** and after exposure to increasing concentrations of Akrinor^TM^ (Akr), theophylline (Theo) and norephedrine (Nor) and after subsequent exposure to noradrenaline (NA) respectively. Concentrations of theophylline and norephedrine matches to the respective concentration in Akrinor^TM^. n/n, indicates number of IMA preparations/number of patients. Experiments were performed in the absence of phenoxybenzamine. **#** indicates *p* < 0.05 compared to maximum effects of NA in the presence of theophylline vs. TMC (one-way ANOVA followed by Bonferroni *post hoc* test).

In human internal A. mammaria preparations, we observed a continuous decline in tension over time not only in TMC but also in preparations exposed to Akrinor^TM^ and theophylline. In contrast norephedrine-treated preparations showed stable tension (*p* < 0.05 vs. TMC, ANOVA, followed by Bonferroni). We would interpret this finding as some evidence for α-adrenoceptor-mediated vasoconstriction (**Figure 5**). However, the effect size of relevant concentrations (30 μM) was modest. Sensitivity to subsequent noradrenaline exposure was not affected by pretreatment with norephedrine. Theophylline (maximum concentration 32 μM) alone did not affect tension (**Figure 5A**), but blunted vasoconstriction to subsequent noradrenaline challenge (**Figure 5B**). It should be noted that Akrinor^TM^ did not evoke any vasoconstriction alone, but shifted the concentration-response for subsequent noradrenaline challenge to the right (from -logEC_50_ 6.18 ± 0.08 to 5.23 ± 0.05 M, *p* < 0.05 vs. TMC, ANOVA, followed by Bonferroni).

## Discussion

Here, we found three pharmacodynamic pathways that could explain the *in vivo* effects of constituents of Akrinor^TM^ and norephedrine, eventually metabolized *in vivo*: direct and indirect sympathomimetic actions and potentiation of PKA-evoked inotropy by PDE-inhibition. The main findings are:

(1) Akrinor^TM^ increased force of contraction in human atrial tissue mediated by direct activation of β_1_-AR.
(2) Akrinor^TM^ increased the potency of FSK for its positive inotropic action similar to theophylline, suggesting relevant cAMP accumulation by PDE-inhibition in cardiomyocytes.
(3) Akrinor^TM^ had no constrictor effect in human internal A. mammaria.

### Pharmacodynamic Profile of Akrinor^TM^ *In Vitro*

#### Heart Muscle

##### Direct sympathomimetic effects

Relevant concentrations of Akrinor^TM^ increased force in human atrial trabeculae in the presence of phenoxybenzamine, indicating a direct sympathomimetic action (**Figure 1**). Effects were completely abolished when β_1_-AR where blocked by CGP 20712A. Mediation of inotropic action of Akrinor^TM^ via β_1_-AR but not β_2_-AR is in line with the known selectivity profile of noradrenaline on β-AR expressed in the human heart (Hoffmann et al., 2004). There is no doubt that α-AR stimulation can evoke positive inotropy in human atrium and ventricle (Steinfath et al., 2000; Grimm et al., 2006). In our experiments application of PBA, a non-selective antagonist at the α-AR precludes any detection of putative α-AR-mediated positive inotropy by Akrinor^TM^. However, Akrinor^TM^ was devoid of any vasoconstriction in IMA preparations, whereas controls showed the expected effect upon noradrenaline (**Figure 5**). From the absence of Akrinor^TM^-induced vasoconstriction, we would expect that Akrinor^TM^ does not stimulate α-AR in vessels, making contribution of myocardial α-AR to Akrinor^TM^ inotropy very unlikely. Next, we calculated whether the noradrenaline content of Akrinor^TM^ could explain the observed positive inotropic effect. The potency based on the noradrenaline content in Akrinor^TM^ (calculated -logEC_50_ ∼5.2 M) was much lower than recently measured for noradrenaline (-logEC_50_ value of 7.06 M; Christ et al., 2014). These data suggest that the potency of conjugated noradrenaline at β_1_-AR is about 100 times less than that of native, unconjugated noradrenaline. This finding may be surprising at first glance, but is in line with clinical experience. Akrinor^TM^ is typically given as a single *i.v.* injection. One ampoule of Akrinor^TM^ contains 10 mg theodrenaline. Since noradrenaline and theophylline have almost the same molecular weight (169 vs. 180 g/mol) one ampoule of Akrinor^TM^ should contain approximately 5 mg noradrenaline equivalent. This is much more noradrenaline than used in clinical practice (typical single dose amounts to 8–12 μg)^1^.

##### Indirect sympathomimetic effects

Direct acting sympathomimetic drugs bind to adrenergic receptors to produce their effects. Indirect sympathomimetic drugs do not bind to receptors, but rather produce effects by increasing neurotransmission at noradrenergic synapses. Many synthetic sympathomimetic drugs are classified as mixed-acting agents. A given agent could exert direct activity in one system and indirect activity in another pharmacological system (Trendelenburg, 1969). Here, we observed robust indirect sympathomimetic effects of unconjugated norephedrine in isolated human atrial trabeculae. The data are consistent with previous data in guinea-pig atria (Trendelenburg et al., 1962) and rat heart showing that noradrenaline re-uptake was inhibited by norephedrine with an IC_50_ of 2 μM (Burgen and Iversen, 1965). Interestingly, indirect sympathomimetic action of norephedrine was lost when conjugated to theophylline. Extensive work was done regarding structure–function relationship for different indirect sympathomimetic agents on uptake 1 showing even small modification can drastically affect affinity to transporters (Levitt et al., 1974). There are no such data available for cafedrine. However, we would expect norephedrine has lost affinity to uptake 1 transporters when conjugated to theophylline. In contrast to the study on guinea-pig atria (Trendelenburg et al., 1962), we could not find evidence for a direct sympathomimetic effect of unconjugated norephedrine, i.e., in the presence of phenoxybenzamine. The reasons are not known, but phenoxybenzamine may inhibit noradrenaline re-uptake more effectively than the *in vivo* reserpine-pretreatment of animals.

#### PDE-Inhibition

Inhibition of PDE slows down enzymatic break down of cAMP and can thereby increase intracellular level of cAMP and mimic and potentiate effects of β-adrenergic stimulation. Akrinor^TM^ consists of theophylline, a non-selective PDE-inhibitor with low potency (IC_50_ 1 mM; Butcher and Sutherland, 1962). Pretreatment of guinea-pig papillary muscles with 200 μM theophylline shifted the concentration-response for the positive inotropic effect of the β-AR agonist isoprenaline by about half a log unit (Korth, 1978). Our results with the direct adenylyl cyclase activator forskolin are in line with those findings. In addition, our results illustrate the small therapeutic window for PDE-inhibition with depressed inotropic responses when higher concentrations were used. Like theophylline, Akrinor^TM^ was able to sensitize human atrial trabeculae to FSK.

Pharmacodynamics of noradrenaline and norephedrine are altered by conjugation to theophylline. Potency of noradrenaline to evoke positive inotropy via activation of β_1_-adrenoceptors is drastically decreased. Indirect sympathomimetic effects of norephedrine are completely lost. In contrast, the ability of theophylline to potentiate FSK effects on inotropy was well-preserved in Akrinor^TM^. Effect size, expressed as shift in sensitivity was about half a log unit and fits nicely to data recently reported for selective inhibition of PDE3 in human atrium and ventricle (Christ et al., 2006; Molenaar et al., 2013). However, effects were restricted to the highest concentrations (420 mg/l). Lower concentrations of Akrinor^TM^ (4.2 and 42 mg/l) did not shift the FSK concentration-response curves, making considerable PDE-inhibition *in vivo* unlikely.

#### Arterial Rings

##### Direct sympathomimetic effects

In TMC we saw the classic vasoconstriction with noradrenaline. Potency and E_max_ of noradrenaline are in line with earlier reports on human IMA (EC_50_ value of 560 vs. 660 nM) (Giessler et al., 2002). In contrast, Akrinor^TM^ when applied alone was devoid of any vasoconstriction, suggesting absence of relevant α_1_-AR-agonistic activity. This may be surprising given that it showed full agonistic activity via β_1_-AR in atrial trabeculae. However, its potency was drastically lower than expected from its noradrenaline content. In addition, the EC_50_ values of noradrenaline for α_1_-AR-mediated vasoconstriction are higher than for β_1_-AR positive inotropy (600 vs. ∼100 nM), and the maximal concentration used in the arterial rings was 168 vs. 420 mg/l in the trabeculae. Yet, based on these calculations we still expected some vasoconstriction at 168 mg/l Akrinor^TM^, containing 19 μM noradrenaline conjugated to theophylline (theodrenaline). In contrast, Akrinor^TM^ shifted the concentration-response curve for the noradrenaline-mediated vasoconstriction by about one log unit to the right, supporting the assumption that noradrenaline in Akrinor^TM^ has lost its agonistic activity at α-AR and behaves as a competitive antagonist. This finding is in line with a very early report about block of α-AR-mediated vasoconstriction in dog A. renalis by cafedrine (Sakai et al., 1969). An alternative explanation would be that the PDE-inhibition in Akrinor^TM^ underlies the effect. Detailed Schild plot analysis for noradrenaline in the presence of Akrinor^TM^ and it constituents could help to clarify that issue.

##### Indirect sympathomimetic effects

From the experiments with atrial trabeculae, we would not expect relevant indirect sympathomimetic effects of cafedrine. Therefore, we exposed IMA preparations to norephedrine only. While we saw maximum inotropic effects with 10 μM in atrial trabeculae, there was only a slight increase in tension in arterial rings with concentrations as high as 300 μM. Norephedrine may act on α_1_-AR as a partial agonist and could thereby behave as an antagonist in the presence of a full agonist (noradrenaline that should be released by norephedrine). However, in earlier work apparent affinity of norephedrine to α_1_-AR was found rather very low (220 μM) (Minneman et al., 1983). Since the -logEC_50_ for subsequent noradrenaline challenge was not drastically lower in the presence of norephedrine than in TMC we would exclude any relevant direct block of α_1_-AR by norephedrine in our experiments. Therefore, we would assume small effect size of norephedrine in IMA related to differences in the anatomical structure of tissue (smaller amount of nerve endings in blood vessel vs. heart muscle), as extensively reviewed (Trendelenburg, 1969).

#### PDE-Inhibition

In vessels, any increase in cAMP favors relaxation and can therefore reduce vasoconstriction upon α_1_-AR stimulation (Morgado et al., 2012). PDE3 does not only hydrolyze cAMP but also cGMP (Maurice et al., 2014; Bobin et al., 2016), a second cyclic nucleotide mediating vasodilation. In our experiments maximum response of α_1_-AR-mediated vasoconstriction in IMA preparations were almost completely depressed by high concentrations of theophylline. This finding is in line with earlier reports showing the selective PDE3 inhibitor milrinone inhibits vasoconstriction by phenylephrine in human IMA and A. radialis preparations (He and Yang, 1996, 2000). In contrast to theophylline, maximum responses to noradrenaline were unchanged by pretreatment with Akrinor^TM^. The finding that theophylline no longer blunts noradrenaline-induced vasoconstriction when conjugated may suggest that loss of PDE-inhibition may be substrate-specific (cGMP), since the effect of Akrinor^TM^ on cAMP-mediated responses (inotropy in atrial trabeculae) are nicely preserved. Further biochemical studies are needed to clarify that issue.

### Cafedrine an Inactive Component of Akrinor^TM^ ? -Pharmacokinetic Considerations

From our *in vitro* findings, one could assume that cafedrine has no major effect at all. We measured cafedrine effects over a large concentration range. We extrapolated a peak concentration of 42 mg/l, resulting from an injection of 210 mg Akrinor^TM^ in 5 l blood, as no further data on Akrinor^TM^ distribution in plasma are available. This should result in cafedrine concentrations of about 100 μM. Cafedrine half-life is about 60 min (Koch and Wenzel, 2006). Exact metabolites are not known. Detailed knowledge of cafedrine pharmacokinetics is the basis of understanding of cafedrine *in vivo* pharmacodynamics. Interestingly and in contrast to catecholamines, maximum effects of cafedrine were observed with some delay but decrease rather slowly (Sternitzke et al., 1984). This finding could indicate that cafedrine, unable to evoke indirect sympathomimetic actions by itself, is metabolized to its active congener norephedrine or other active metabolites. In addition, it seems conceivable that theodrenaline could be also metabolized to noradrenaline. Interpretation about Akrinor^TM^ as a pro-drug are at present pure speculation. Further pharmacokinetic studies should help to clarify the contribution of cafedrine to the overall effect of Akrinor^TM^ and better understand the time course of effect of Akrinor^TM^.

## Conclusion

Among agents used for blood pressure stabilization Akrinor^TM^ is unique because of its prolonged duration of action and its neutral effect on vascular resistance. These experimental results could help support clinical decision-making, which substance to choose for the treatment of anesthesia-induced hypotension in patients with comorbidities (i.e., coronary artery disease). On-going clinical trials should not only help to clarify the clinical usefulness of Akrinor^TM^ but also help to elucidate whether prolonged action of Akrinor^TM^ may result from being a pro-drug.

### Limitations

The direct and indirect effects of Akrinor^TM^ and some of its constituents described in this study do not allow direct interpretation of the effects of the drug *in vivo*. Any intervention that affects the uptake or storage of noradrenaline could increase noradrenaline concentration in the systemic circulation and evoke effects in organs with limited storage capacity for noradrenaline. For example, clinically relevant concentrations of aminophylline can increase plasma concentrations of epinephrine and to a lesser extent noradrenaline in humans (Vestal et al., 1983). While the potencies of noradrenaline to evoke inotropy and sensitivity to PDE-inhibition are very similar in human ventricular and atrial tissue (Christ et al., 2006; Molenaar et al., 2013), the effect size of indirect sympathomimetic agents like norephedrine and cafedrine may differ. Vascular resistance is regulated by arterioles and not by arteries investigated here. However, we cannot access to human arterioles. Effect size of indirect sympathomimetic activation demonstrated here may reflect noradrenaline stored within the tissue under investigation. Finally, while our data suggest reduced binding affinities of noradrenaline and norephedrine when conjugated to theophylline direct binding data are lacking. During the last decades investigation of the principles of indirect sympathomimetic drug action has lost some attraction. Therefore, we had to refer to older papers. Some of them may no longer represent state of the art. Nevertheless, the principles of indirect sympathomimetic actions still represent textbook knowledge (Westfall and Westfall, 2011). Probably there is a continuum of activity from predominantly direct-acting to predominantly indirect-acting drugs. Thus, this classification has to be interpreted as a relative rather than absolute one (Westfall and Westfall, 2011).

## Author Contributions

BK, SP, EM, YS, and K-DS performed research. SS, TE, BK, HR, ES, RB, and TC planned experiments. BK, SP, YS, EM, K-DS, HR, ES, RB, and TC analyzed results. BK, ES, HR, TE, TC, and SS wrote the manuscript. All authors approved the final version of the manuscript.

## Conflict of Interest Statement

TEVA GmbH, Ulm, Germany, funded this study. TC and SS have received speaker honoraria from TEVA ratiopharm GmbH, Ulm, Germany. The other authors declare that the research was conducted in the absence of any commercial or financial relationships that could be construed as a potential conflict of interest.

## Footnotes

1 http://www.drugs.com/pro/norepinephrine.html

## Supplementary Material

FIGURE S1 **Effects of theophylline and Akrinor^TM^ on concentration-response curves for the positive inotropic effect of forskolin**. Mean values ± SEM of force under control conditions (300 nM CGP 20712A; CGP), in the presence of different concentrations of theophylline (Theo, top) or Akrinor^TM^ (Akrinor, bottom) and after subsequent exposure to increasing concentrations of FSK. n/n indicates number of trabeculae/number of patients.

## Abbreviations

FSK: forskolin
IMA: internal mammary artery
PDE: phosphodiesterase
PKA: protein kinase A
TMCs: time-matched controls.
