# Supplementary figures and images for: AkrinorTM, a Cafedrine/ Theodrenaline Mixture (20:1), Increases Force of Contraction of Human Atrial Myocardium But Does Not Constrict Internal Mammary Artery In Vitro
Source: Front Pharmacol. 2017 May 23;8:272. doi: 10.3389/fphar.2017.00272 (PMC5441130; doi:10.3389/fphar.2017.00272)

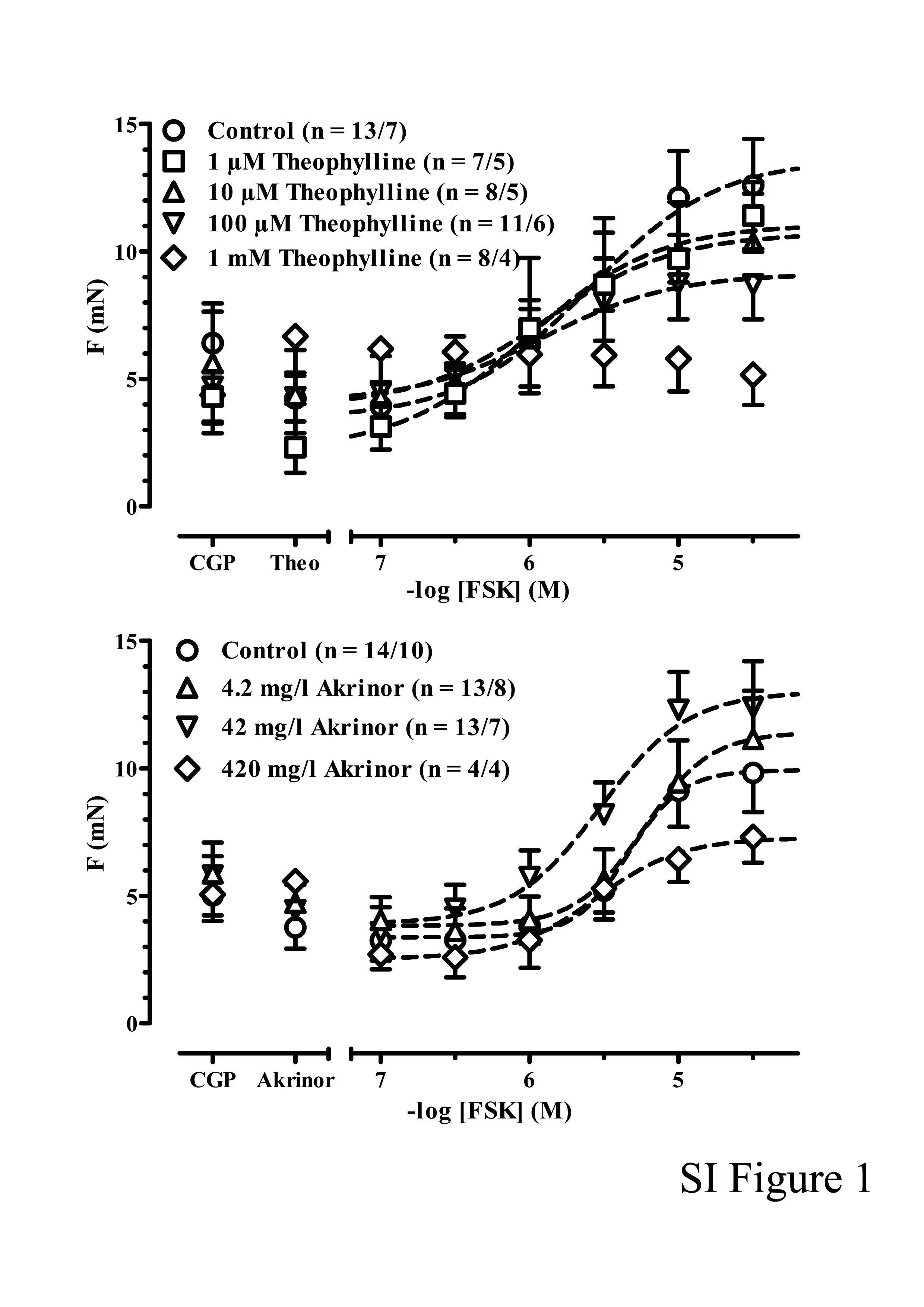

Supplement: FIGURE S1 — Effects of theophylline and AkrinorTM on concentration-response curves for the positive inotropic effect of forskolin. Mean values ± SEM of force under control conditions (300 nM CGP 20712A; CGP), in the presence of different concentrations of theophylline (Theo, top) or AkrinorTM (Akrinor, bottom) and after subsequent exposure to increasing concentrations of FSK. n/n indicates number of trabeculae/number of patients. [file Image_1.TIF]
