# Supplementary material for: AkrinorTM, a Cafedrine/ Theodrenaline Mixture (20:1), Increases Force of Contraction of Human Atrial Myocardium But Does Not Constrict Internal Mammary Artery In Vitro
Source: Front Pharmacol. 2017 May 23;8:272. doi: 10.3389/fphar.2017.00272 (PMC5441130; doi:10.3389/fphar.2017.00272)
Supplement: Supplementary file 2 [file Table_1.DOCX]

**SI Appendix; Table 1 Patient characteristics**

|  |  | |  |
| --- | --- | --- | --- |
| N | 32 |  | |
| Gender [m/f] | 23 / 9 |  | |
| Age [years] | 64.5 ± 1.7 |  | |
| BMI [kg/m^2^] | 27.6 ± 0.9 |  | |
| Sinus rhythm | 32 |  | |
| Hypertension, n | 25 |  | |
| Diabetes mellitus, n | 5 |  | |
| Hyperlipidaemia, n | 10 |  | |
| CAD, n | 20 |  | |
| AVD/MVD, n | 10 |  | |
| CAD + AVD/MVD, n | 2 |  | |
| LVEF [%] | 54.9 ± 1.7 |  | |
| Cardiovascular medication (n) |  |  | |
| Digitalis | 0 |  | |
| ACE-Inhibitors | 18 |  | |
| AT_1_-blockers | 7 |  | |
| β-blockers | 21 |  | |
| Ca^2+^-channel-blockers | 10 |  | |
| Diuretics | 6 |  | |
| Nitrates, n | 3 |  | |
| Lipid-lowering drugs | 20 |  | |

Abbreviations: AT, angiotensin receptor; AVD, aortic valve disease; CAD, coronary artery disease; LVEF, left ventricular ejection fraction; MVD, mitral valve disease; SR, sinus rhythm.
